# Monitoring of Processing Conditions of an Ultrasonic Vibration-Assisted Ball-Burnishing Process

**DOI:** 10.3390/s20092562

**Published:** 2020-04-30

**Authors:** Aida Estevez-Urra, Jordi Llumà, Ramón Jerez-Mesa, Jose Antonio Travieso-Rodriguez

**Affiliations:** 1 Department of Mechanical Engineering and Manufacturing, Universidad de Sevilla, 41092 Sevilla, Spain; aeurra@us.es; 2 Department of Materials Engineering, Universitat Politècnica de Catalunya, 08019 Barcelona, Spain; jordi.lluma@upc.edu; 3 Department of Engineering, Universitat de Vic, Universitat Central de Catalunya, C. de la Laura 13, 08500 Vic, Barcelona, Spain; 4 Department of Mechanical Engineering, Universitat Politècnica de Catalunya, 08019 Barcelona, Spain; antonio.travieso@upc.edu

**Keywords:** accelerometer, process monitoring, natural frequencies, ball burnishing, ultrasonic, piezoelectric, surface integrity

## Abstract

Although numerous references present the beneficial effects on surface integrity of ultrasonic vibration-assisted ball burnishing (UVABB), nothing has been reported about the dynamic behavior of the UVABB tool, workpiece, and machine triad during the process. In this paper, a dynamic monitorization through a set of 5 accelerometers is tested to analyze the interactions between the tool–workpiece–machine mechanical assembly. A UVABB tool attached to a milling machine and equipped with a piezoelectric stack that is able to assist the process with a 40-kHz vibration is tested on a milled C45 steel surface. First, the natural frequencies of the mechanical system are obtained through hammer impact tests. Then, the vibratory signals transmitted during the execution of the process are monitored and compared to those: two feed velocities and two burnishing preloads, all with and without vibration-assistance. Results show that the proposed accelerometer set is valid to assess the behavior of a UVABB process. The system’s natural frequencies are not varied by vibration-assistance and are not excited when the piezoelectric is functioning. It is confirmed that UVABB is safe for the machine and the tool, and there is no unexpected excited frequencies due to the piezoelectric excitation.

## 1. Introduction

The high standards that today’s industry demands from workpieces has had no precedents in history due to the high competitiveness present in key sectors for economical development. Mechanical components from machine-tools, automobiles, aircraft, trains, moulds, and many other industrial elements are clear examples of workpieces on which excellence must be searched in terms of surface integrity or geometrical tolerance [1]. More specifically, an adequate surface roughness, elevated surface hardness and high compressive residual stress fields are basic to guarantee a long lifespan of those parts, as well as to avoid unexpected failures when subjected to cyclic stress.

Ball burnishing is a finishing process that is highly extended nowadays because of its robustness and its capability to provoke a comprehensive effect on surface integrity (i.e., improvement of the triplet roughness–hardness–residual stress) [2]. The operation consists of plastically deforming the irregularities of the target surface by means of a controlled force transmitted by a sphere [3]. Its versatility to treat concave and convex surfaces has supported its expansion in the industry [4]. However, scientific and technical knowledge has focused historically on its conventional version, namely, the non-vibration-assisted ball burnishing (NVABB) [5]. However, the last years have witnessed the extension of a second variant, consisting of accompanying the ball during its displacement with an alternative oscillation in the perpendicular direction. This version of the process is referred to as vibration-assisted ball burnishing (VABB) [6].

What is the interest of assisting the process with an additional vibration? Kozlov et al. (1995) [7] explained that the yield strength of a material can change if, while experimenting plastic deformation, an exogenous vibratory source causes a variation in the driving force magnitude. This variation of the elastoplastic properties of a material is called acoustoplasticity [8]. The scientific interpretation of this effect is that the subsequent wave which is transmitted through the material’s structure as a consequence of the external vibration enhances the mobility of dislocations inside the crystal lattice and, therefore, increases the capacity of plastic deformation observed macroscopically. This causes a decrease of the yield strength and, as a consequence, eventually enables further deformation with lower external forces. Therefore, assisting the ball-burnishing process through a vibration contributes to obtaining better results with regards to its conventional version [9].

Numerous past studies can be found giving experimental evidence that acoustoplasticity can be effectively applied to improve manufacturing processes on different alloys such as carbon steel [10], magnesium [11], or aluminum alloys [12], as well as pure materials such as 99.99% pure copper [13]. For instance, Jung and Siang (2008) [10] proposed the introduction of ultrasonic vibration by means of a piezoelectric stack attached to a polishing tool to assist the process itself on a mold steel alloy. As a consequence, the average surface roughness $R_{a}$ decreased to 0.036 μm, while the nonassisted process resulted in a higher value: 0.100 μm. Furthermore, the ball used for the process experienced 28% less wear. The introduction of vibration-assistance in machining processes has also proved to be a satisfactory practice on alloys such as SUS304 stainless steel or cuprous alloys, as many researchers highlight [14,15,16]. The effects of ultrasonic-vibration-assistance are linked to a conspicuous decrease of machining forces and chip thickness, which results in lower chatter and higher stability of the process. The consequence is surfaces showing lower surface roughness and an increase in the lifespan of machining inserts.

Numerous systems are used to deliver vibration-assistance into manufacturing processes [1], but around 80% of them use similar systems as the one object of study in this paper: 20- or 40-kHz resonant systems with low amplitude movement—from 3 to 20 μm [17]. By making the system resonant, the stability of the vibratory behaviour is stabilized in time. Indeed, Babitski et al. [18] observed that systems that could work under different amplitude regimes showed unstable behaviour, and that caused a fall of almost 50% in the final surface roughness obtained. This kind of linear vibratory system, ideal for transmitting a high-frequency 1D oscillating movement, is based on a slender sonotrode on whose tip the burnishing ball or the machining insert is installed. Its design must be robust enough to guarantee an overall tool rigidity to satisfy two conditions. First, to prevent the tool from excessively deforming under bending stress (in lathe setups, where the tool works as a cantilever) or by buckling and bending in milling machine setups. Secondly, undesired transverse vibrations must be avoided. Indeed, a combination of these effects could lead the process to actually harm the surface [19] and derive in a higher surface roughness than expected [14,15].

**Figure 1:**
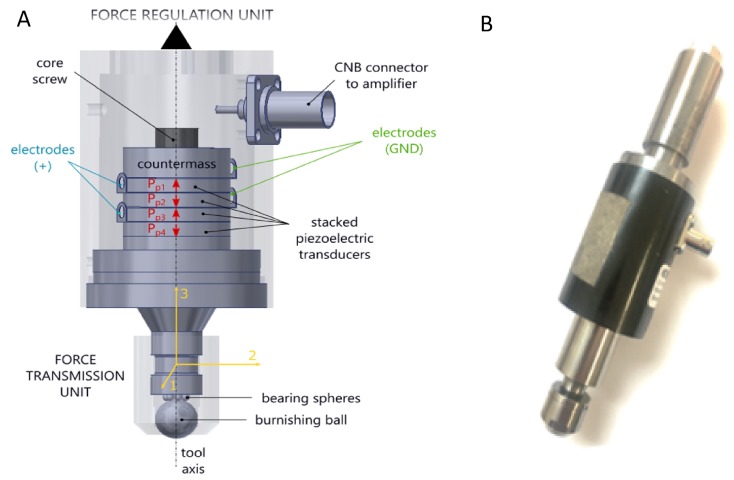
Ultrasonic vibration-assisted ball-burnishing (UVABB) tool equipped with a 1D linear displacement system through piezoelectric stack designed by Jerez-Mesa et al. (2018) [20]. (**A**) General scheme of the piezoelectric system; (**B**) Real image of the prototype used for vibration monitoring.

The ultrasonic-vibration-assisted ball-burnishing (UVABB) process has been extensively reported by Jerez-Mesa et al. (2018) [20]. This tool works so that vibrations are introduced through a sonotrode at whose tip the burnishing ball is installed, and whose length changes as an effect of thickness variation of a piezoelectric stack subjected to a difference of potential [21]. This tool is used for the monitoring and sensor installation during the works reflected in this paper. Figure 1 shows the design referred to. Its characterization lead to the conclusion that the working conditions (precharge or feed velocity during the process) are not influenced by the active resonant frequency of 40 kHz. For that reason, in this study, vibrations are monitored up to a maximum frequency of 24 kHz as transmitted by the tool, or 5 kHz on the workpiece.

Monitoring the condition of the UVABB tool during the application of the process is vital to obtain good results on the target workpiece [22]. In fact, guaranteeing that the effect of the vibratory movement is fully concentrated on the process itself is basic to ensure that there are no setbacks that could affect the operation, or hinder the effect of vibration-assistance. This could lead to a variety of consequences, from energy waste to surface integrity hindering. Therefore, with the aim of guaranteeing that this takes place, this paper presents the characterization of the machine–tool–workpiece ensemble to determine the natural frequencies of these elements, as well as identify other harmonic components forced by the process’s progress. In UVABB, the source of excitation could be the different dynamic elements composing the machine itself, or the excitation module that takes part of the tool. If the machine’s or tool’s natural frequencies were to be coincidental with these excitation sources, undesired and unfavorable couplings could occur between resonant states.

**Figure 2:**
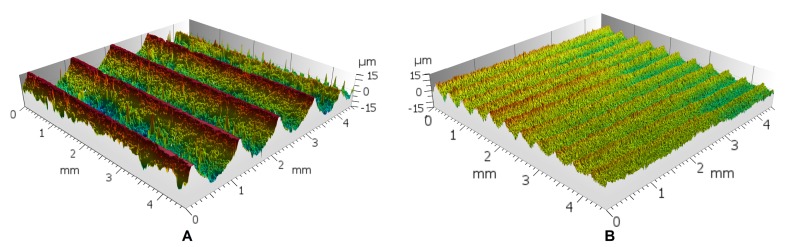
Surface texture modification through UVABB with the prototype object of study—after results obtained previously by Jerez-Mesa et al. (2018). (**A**) Before UVABB; (**B**) After UVABB [9].

In this paper, a prototype whose effectiveness has been confirmed in previous experiments on a similar carbon steel alloy (Figure 2) is the object of study [9]. Therefore, the monitorization that is featured in this paper is fundamental to know in-depth the phenomena occurring during the UVABB process that could affect the results. Furthermore, it is also its aim to understand whether the interaction of the burnishing ball with the original surface could lead to secondary excitations and cause tool malfunctions. Therefore, the knowledge generated here is of utter importance for industries (i.e., transportation, biomedical, etc.) interested in incorporating the UVABB in their production lines. Currently, the authors have not found previous bibliographical references dealing with this kind of monitoring and modal study of a process where so many excitation sources are present.

## 2. Materials and Methods

### 2.1. Experimental Setup

To characterize the machine–tool–workpiece ensemble, impact tests were performed. Additionally, the signals transmitted in different directions were monitored during the application of the UVABB process. The UVABB tool was installed on a CNC LAGUN MC600 milling machine (LAGUN Machinery Ltd., Vitoria, Spain), and the target workpiece was fixed on the milling table. With this design, the rigidity conditions of the assembly are only influenced by the variation of the parameters that are objects of study, namely, the burnishing preload and the vibration assisting the process.

The workpiece is constituted by a 100 mm × 80 mm × 60 mm hot-rolled C45 (according to the UNE-EN 10027-1:2017 standard) steel block with a Vickers hardness of 242 ± 5 HV1. The surface to be burnished was prepared by a previous milling operation with an 8-mm-diameter hemispherical tool, consisting of adjacent passes with a 0.7-mm offset. The cutting velocity was 400 m/min, and 800 mm/min was the selected feed velocity. The result is a periodic peak–valley surface showing an average roughness $R_{a}$ of 17.7 μm. The ulterior burnishing operation was invariably performed perpendicular to this machining direction.

**Figure 3:**
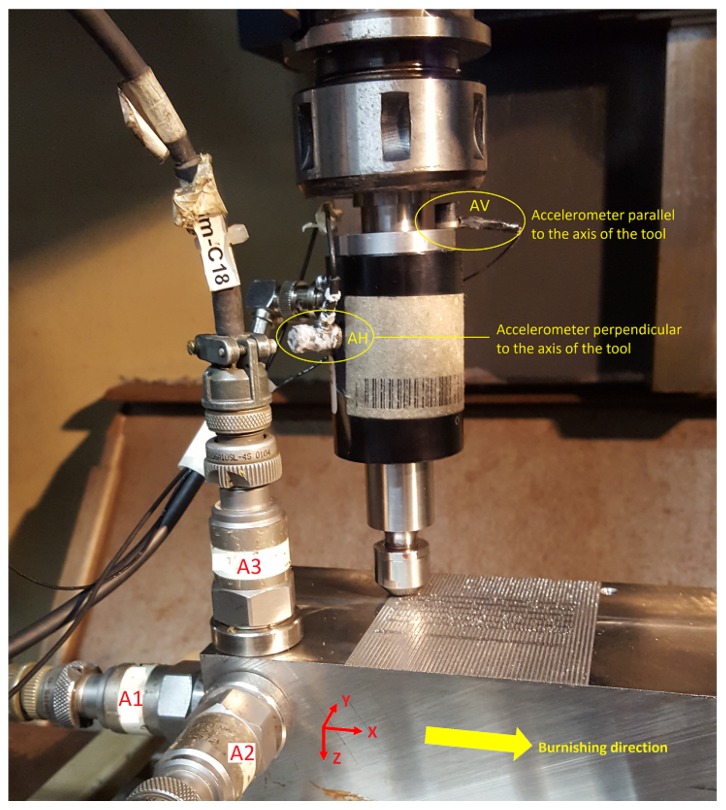
Experimental setup of accelerometers on the workpiece (A1, A2, and A3) and on the tool (AV and AH).

To monitor the vibrations, 5 accelerometers (Table 1) were installed: 2 on the tool and 3 on the workpiece. The overall setup is shown in Figure 3.

### 2.2. Monitoring of the UVABB Force

The actual load transmitted by the UVABB to the target surface is governed by the compression of a spring installed inside the tool-holder [20]. As the tool is fixed to the milling machine spindle, this compression is proportional to its vertical posFnew Fiuition and, ultimately, the plunging coordinate programmed in the CNC routine. In this case, different tests were conducted with two different vertical coordinates *Z*—namely, −0.4 and −3.3 mm—that can be translated into nominal preload values of 250 and 400 N, respectively.

This force was monitored during all tests with a dynamometric table KISTLER 9129AA (Kistler Instrumente AG, Winterthur, Switzerland), on which the workpiece was rigidly mounted. The resulting transmitted force signals were acquired through a KISTLER 5070A12100 amplifier (Kistler Instrumente AG, Winterthur, Switzerland). All of them were processed to compute the mean and maximum force attained during all tests, to confirm the stability of the process and guarantee the nonvariation of the rigidity conditions.

### 2.3. Monitoring of Vibrations during UVABB

The characterization of the rigid system composed by the combination of the tool pressing the workpiece’s surface during the process was carried out by performing different tests varying different process parameters, as shown in Table 2. The objective was to determine whether the basic process descriptors could affect the frequency response of the mechanical system or, on the contrary, whether they only depended on its configuration.

- Feed velocity during linear tool displacement $v_{f}$.
- Nominal preload force $F_{p}$. This term refers to the amount of force excerted due to the precompression of the tool with the milling machine of the surface, consequence of spring compression. It is expressed like this to differentiate it to the actual burnishing force, which can vary during the NVABB and UVABB process due to different sources.
- Number of passes on the same target surface *n*.
- Activation of the vibrations (ON) or not (OFF), i.e., vibration-assisted process or not, respectively.

### 2.4. Impact Tests

The natural frequencies of a mechanical system can be determined by obtaining its frequency response signal that results when it is excited by means of a controlled source. Both continuous (sinusoidal or stochastic) and transient excitation can be applied to obtain that signal. In the last case, the obtained signal only lasts during short times. Of all possible methodologies to undertake this action, the impact through an instrumentalized hammer was chosen. This technique is more simple and easy to apply, but it was mostly chosen due to the fact that it allows the user to excite the system in different directions and positions that could have been unreachable if other methods were chosen [23]. Furthermore, it guarantees that no load effect affects the results.

The impact tests were undertaken with a KISTLER 9722A2000 impact hammer (Kistler Instrumente AG, Winterthur, Switzerland) with a steel tip 9902A. The maximum excitation frequency of this device is 9.3 kHz and the maximum force is 11 kN. These descriptors are very adequate, as previous works evidenced that the natural frequency of this kind of tool is in no case higher than 5 kHz [24]. The excitation was performed by applying a rectangular window, and an exponential window was used for the response analysis. A total of 6400 lines were taken for a 1-Hz resolution.

**Figure 4:**
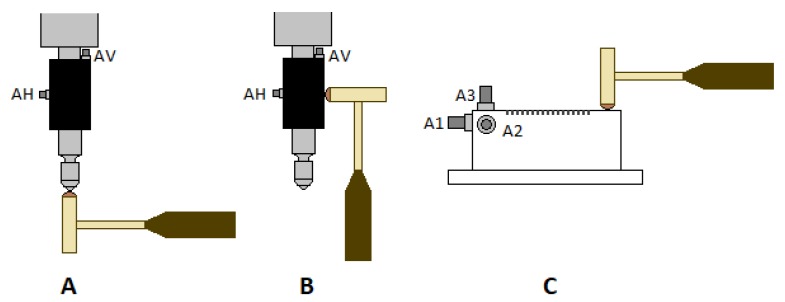
Schemes showing all types of performed impacts. (**A**) Vertically on the tool; (**B**) Horizontally on the tool; (**C**) Vertically on the workpiece attached to the dynamometric table.

Both the workpiece and the tool were impacted in different directions, as is shown in Figure 4. In addition, each test was undertaken under various circumstances, as is reflected in Table 3. It is obvious that vertical impacts on the tool can only be performed if it is not loaded on the workpiece’s surface, whereas the setup enables the test operator to perform the tests under different loading conditions. Like that, it can be quantified how the natural frequency changes with regards to the overall system rigidity.

Ulterior measurements and analyses were performed with a compact data acquisition Brüel & Kjær 3053-B-120 and the PULSE Reflex software, respectively. Six different acquisition channels were used, five of them for the respective measurement points shown in Figure 3 and another one for the input signal generated by the impact with the hammer. Signal acquisition was performed utilizing a Hanning window, with a maximum frequency of 6400 Hz and 51,200 lines with a resolution of 0.125 Hz.

## 3. Result Discussion

### 3.1. Impact Tests

**Figure 5:**
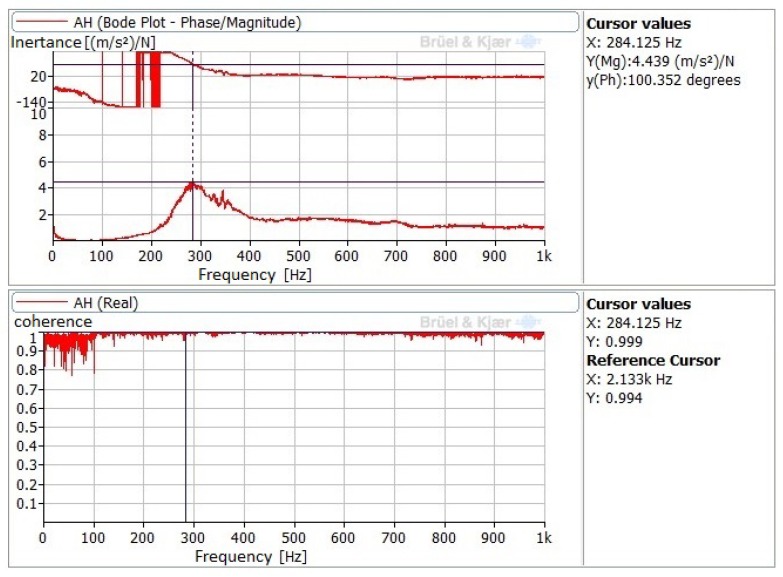
Frequency response functions (FRF) and Bode diagram obtained by AH accelerometer after impact test on the UVABB tool: unloaded and vibrations ON.

The natural frequencies found for the UVABB tool, and resulting from processing the signal registered by accelerometers AH and AV (Figure 4A,B), are presented in Table 4. Values not included in the table are missing due to the fact that they were not excited during the tests. These values evidence that, by changing the load exerted on the workpiece surface changes the rigidity of the system and, therefore, its natural frequencies. However, it can also be seen that these values are not sensitive to piezoelectric excitation, i.e., there is no modal change during a UVABB process compared to an NVABB operation. All values were deduced from the frequency response functions (FRF) and Bode diagrams to confirm coherence between the input signal generated by the hammer and the corresponding FRF. Figure 5 shows one as an example.

**Figure 6:**
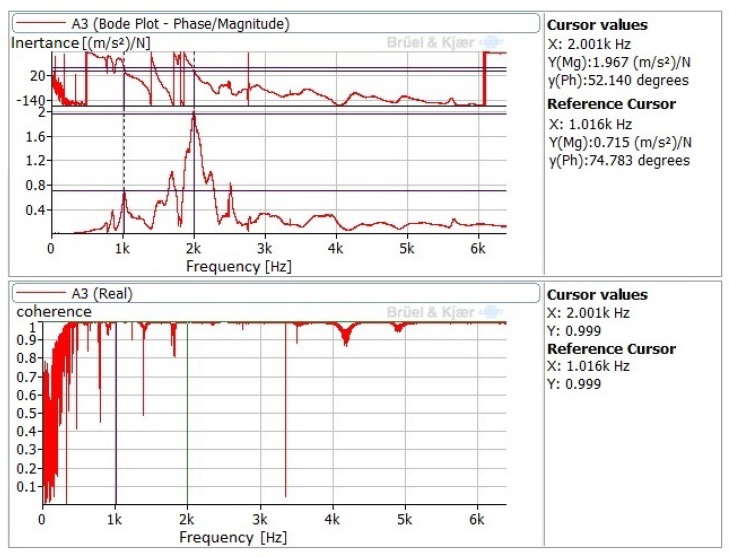
FRF and Bode diagram obtained by A3 accelerometer (vertical direction) after impact test on the unloaded workpiece.

On the other hand, natural frequencies measured on the workpiece are presented in Table 5. These are the result of processing signals acquired by accelerometers A1, A2, and A3 installed on the workpiece’s surface (Figure 4C), such as the FDF and Bode diagram shown in Figure 6. Once again, the results demonstrate the invariability of natural frequencies of the mechanical system composed by the machine–tool–workpiece triplet, regardless of the preload level or the situation of the piezoelectricity in terms of excitation.

Furthermore, the magnitude of the obtained natural frequencies are much lower than the hammer’s maximum excitation frequency and also the resonant frequency on which the functioning of the UVABB tool is based—being less than 20% from that value. After all the results explained above, it can be asserted that the performance of the UVABB process is robust in terms of dynamic response of the whole system, and does not change with the loading conditions or the external excitement of the piezoelectric stack for which the UVABB accounts for.

### 3.2. Vibration Monitoring

The vibratory signals acquired during the ball-burnishing tests were processed and analyzed in both time and frequency domains. In those signals, no components with frequencies higher than 6 kHz were detected. Therefore, that value was taken as a threshold to analyze the results and evidenced that there are significant differences between vibrations measured on the workpiece and on the UVABB tool, as is explained in the following paragraphs.

**Figure 7:**
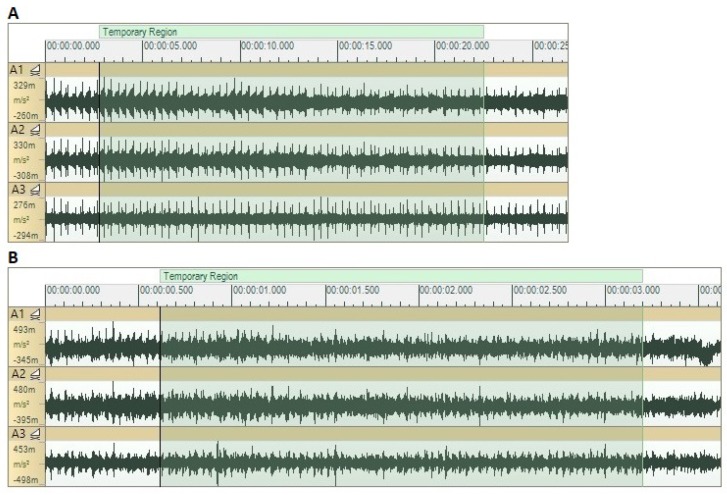
Signals measured on the workpiece during the UVABB process with a 250-N nominal preload in all three spatial directions. (**A**) 90 mm/min; (**B**) 900 mm/min.

The signals acquired on the workpiece through accelerometers A1, A2, and A3 in the time domain for both tested feed velocities evidenced the presence of periodic impacts that derive in a similar time behavior in all three spatial directions (Figure 7). However, the signal corresponding to the 900 mm/min test presents more random components, so the periodicity of those impacts is more difficult to determine.

**Figure 8:**
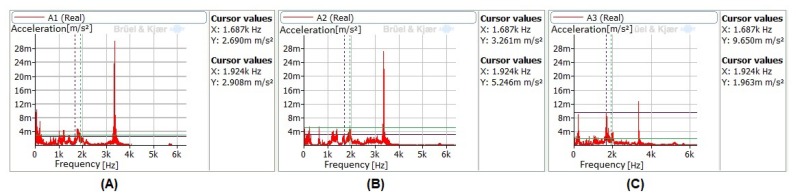
Signals showing natural frequencies of the workpiece being burnished at 900 mm/min, 400 N, 3 passes, and vibrations ON. (**A**) Accelerometer A1; (**B**) Accelerometer A2; (**C**) Accelerometer A3.

The spectral analysis of those time signals can be associated to the functioning of the milling machine and are present since it is turned on. Its amplitudes are higher in both longitudinal directions *X* and *Y* (accelerometers A1 and A2), with values that almost double the signal recorded in the vertical direction *Z* (accelerometer A3), as can be seen in Figure 8. It can also be appreciated that the natural frequencies are indeed excited between 1000 and 2000 Hz, as was already shown in Table 5.

**Figure 9:**
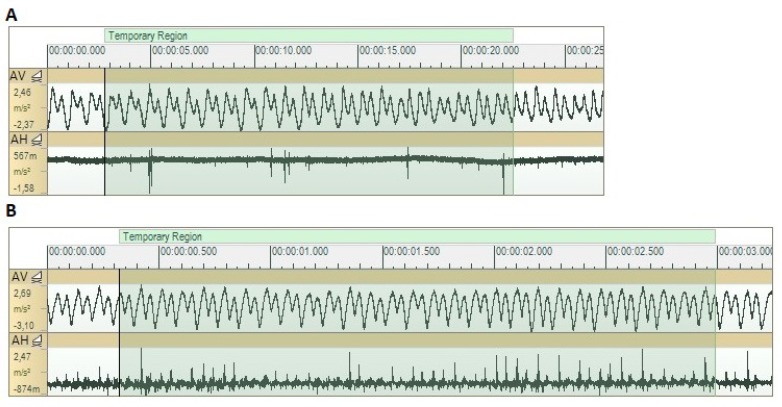
Signals measured on the UVABB tool along its vertical and transverse directions, during tests with vibrations ON. (**A**) 90 mm/min, 250 N, 1 pass. (**B**) 900 mm/min, 400 N, 3 passes.

The vibratory signals acquired on the tool are very different from the ones acquired on the workpiece, these are presented above. Indeed, the signals acquired along the vertical direction of the tool (accelerometer AV) is highly periodic and remains the same regardless of the testing conditions, unlike the signals recorded along the horizontal direction (accelerometer AH) that evidence different behavior depending on the feed velocity of the test (Figure 9). Tests performed with a 900 mm/min feed velocity show that their periodicity fits more exactly with the periodic repetition of its vertical signal’s correspondents.

**Figure 10:**
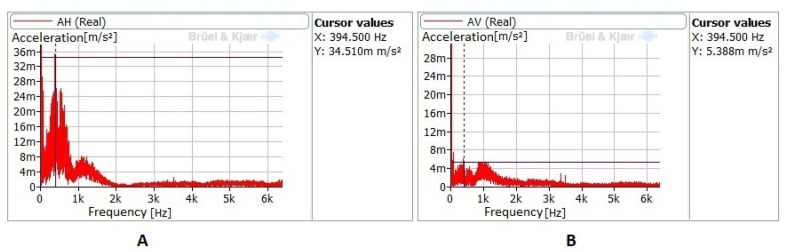
UVABB tool excitation signals measured on the longitudinal and radial directions. 900 mm/min, 400 N, 1 pass, vibrations OFF. (**A**) AH sensor; (**B**) AV sensor.

The spectral analysis of the same signals measured on the tool show that its natural frequencies, measured during the preliminary impact tests, are indeed excited during the process, and that excitation is higher when a 900-mm/min feed velocity is selected to perform the process. For instance, Figure 10 shows the spectrum obtained from the signals recorded by the AH and AV accelerometers, depicting those excitation zones around 394 Hz and 1000 Hz that constitute the natural frequencies of the system. Amplitudes are higher in the horizontal measurements than in the vertical ones.

The presented results take us to the conclusion that during the ball-burnishing process performed with an UVABB tool, whether transmitting vibrations or not, the natural frequencies of that tool are actually excited. However, there is no evidence that it interferes with the interaction of the tool and the workpiece, or the vibratory signal that the former transmits to the latter. The signal arriving to the workpiece seems to change depending on the feed velocity with which the process is performed, as the period of the repetitive signal changes in the time domain. However, it is only a consequence of the process’ kinematics, and does not affect its effectiveness.

The higher amplitudes of the signals measured by accelerometers A1 and A2 are justified by the interference that the workpiece’s surface performs on the tool as it moves on it to perform plastic deformation. In the vertical direction (accelerometer A3), that movement is constrained by the compression load of the UVABB on the tool, and for that reason it presents a lower magnitude.

### 3.3. Monitoring of the Burnishing Force

**Figure 11:**
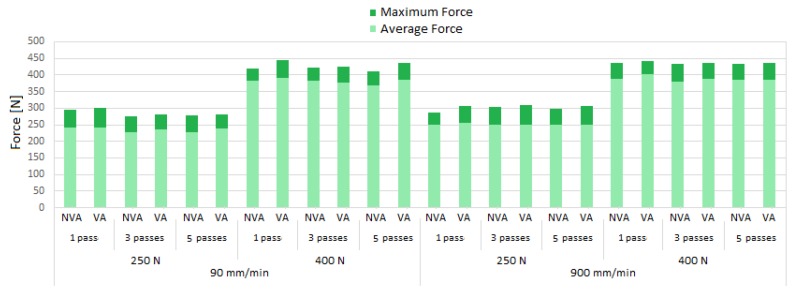
Average and maximum burnishing forces registered during all tests.

The acquisition and processing of the burnishing forces during all tests confirm that the average preloads that were exerted were around the nominal values of 250 and 400 N (Figure 11). The forces prove to be relatively stable, regardless of the testing conditions. This observation is more clear in the tests performed with the highest feed velocity. For 90 mm/min tests, the maximum force variation is 8%.

**Figure 12:**
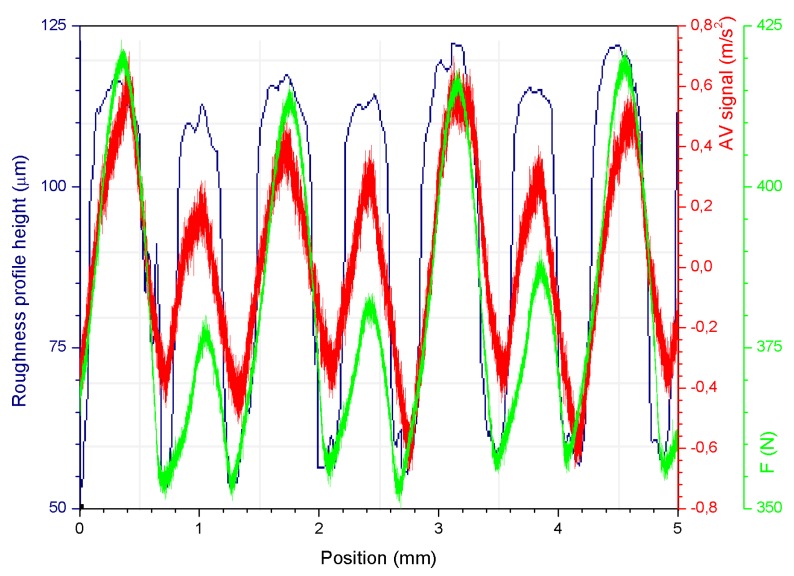
Vibratory signal acquired by the AV accelerometer (red), roughness profile (blue), and burnishing force (green) variation registered during the VABB process—90 mm/min, 400 N, and 5 passes—.

This variation is quite natural during the application of this kind of ball-burnishing test, and does not negatively affect the results. As highlighted previously by Jerez-Mesa et al. (2018), the source of change could be attributed to the absorption of the surface irregularities by the spring inside the tool during the process [9]. However, until now, no physical explanation had been formally found to explain the fact. Here, a clear correspondence of the signal acquired by the vertical accelerometer of the tool (AV), the burnishing force variation, and the topological profile on which the ball rolls during the experiment, confirms the hypothesis exposed before (Figure 12). On the other hand, these points also allow the researchers to confirm that the low-frequency vibrations observed during the process have an intrinsic nature and depend mostly on the type of roughness that the original surface presents.

## 4. Conclusions

In this paper, a monitoring setup composed of five accelerometers and a dynamometric table is proposed to monitor and evaluate the dynamic response of triad formed by an UVABB tool, milling machine, and C45 steel workpiece. The proposed structure has proved to succeed in characterizing the whole system in terms of the vibratory signals transmitted through the mechanical elements during all kind of tests—vibration-assisted or not, and with different burnishing preloads. Furthermore, it has been proven that the natural frequencies of the tool and machine are not excited by the vibratory signals deployed during these tests. Finally, it has been found that the source of variation of the burnishing force—that should in theory be constant due to the fact that it depends on the compression of a spring inside the tool at a macro level—has a micrometrical origin, to the extent that the frequency variation of the force is coincidental with the roughness height variation of the profile being treated.

These results are fundamental to defend the implementation of the UVABB process in productive lines, with no risk of harming the hardware or provoking unexpected effects on the systems sharing the manufacturing layout. Furthermore, it shows that the introduction of an ultrasonic vibration-assistance does not harm the process or jeopardize the results that could be obtained from it.

## Figures and Tables

**Table 1:** Accelerometer set used in the sensorization of the machine–tool–workpiece setup.

Measurement Direction	Accelerometer	Frequency Range (Hz)	Weight (g)
Parallel to tool axis (AH)	MMF Type KS91B	0.3–30,000	1
Perpendicular to tool axis (AV)	Brüel and Kjær Type 4397	1–25,000	2.4
Workpiece’s *X*, *Y*, and *Z* directions	KISTLER Type 8752A50	0.5–5000	115

**Table 2:** Parameters varied and combined during different burnishing tests.

$F_{p}$ (N)		$v_{f}$ (mm/min)		Vibration		*n*		
250	400	90	900	ON	OFF	1	3	5

**Table 3:** Conditions under which all impact tests were performed.

Impacted Element	Direction	Preload Force $F_{p}$ (N)	Piezoelectric Transducer
UVABB Tool	Vertical	Unloaded	OFF
			ON
	Horizontal	Unloaded	OFF
			ON
		250	OFF
			ON
		400	OFF
			ON
Workpiece	Vertical	Unloaded	OFF
		400	OFF
			ON

**Table 4:** Natural frequencies obtained through impact tests on the UVABB tool.

Testing Condition		1st Frequance (Hz)	2nd Frequance (Hz)	3rd Frequance (Hz)
$F_{p}$ (N)	Vibration			
Vertical impacts				
Unloaded	OFF	519	1471	-
	ON	519	1490	-
Horizontal impacts				
Unloaded	OFF	284	-	-
	ON	584	-	-
250 N	OFF	390	-	-
	ON	390	510	-
400 N	OFF	407	645	4257
	ON	410	635	4231

**Table 5:** Natural frequencies obtained through impact tests on the workpiece through vertical impacts and machine-tool on.

Measurement Direction	Accelerometer	Measuring Condition		1st Freq. (Hz)	2nd Freq. (Hz)
		$F_{p}$ (N)	Vibration		
Burnishing direction (X)	A1	Unloaded	-	1018	1766
	A2	400 N	OFF	1017	1761
	A3		ON	1017	1758
Perpendicular to burnishing direction (Y)	A1	Unloaded	-	1017	1899
	A2	400 N	OFF	1017	-
	A3		ON	1017	-
Vertical (Z)	A1	Unloaded	-	1016	2001
	A2	400 N	OFF	1017	2007
	A3		ON	1017	2000

## Abbreviations

The following abbreviations are used in this manuscript:

FRF	Frequency response function
NVABB	Non-vibration-assisted ball burnishing
UVABB	Ultrasonic vibration-assisted ball burnishing
VABB	Vibration-assisted ball burnishing

## References

[1] Travieso-Rodriguez J.A., Gomez-Gras G., Dessein G., Carrillo F., Alexis J., Jorba-Peiro J., Aubazac N. (2015). Effects of a ball-burnishing process assisted by vibrations in G10380 steel specimens. Int. J. Adv. Manuf. Technol..

[2] Yen Y., Sartkulvanich P., Altan T. (2005). Finite element modeling of roller burnishing process. CIRP Ann..

[3] Gomez-Gras G., Travieso-Rodriguez J.A., Jerez-Mesa R., Lluma-Fuentes J., de la Calle B.G. (2016). Experimental study of lateral pass width in conventional and vibrations-assisted ball burnishing. Int. J. Adv. Manuf. Technol..

[4] Travieso-Rodríguez J.A., Dessein G., González-Rojas H.A. (2011). Improving the surface finish of concave and convex surfaces using a ball burnishing process. Mater. Manuf. Process..

[5] Hiegemann L., Weddeling C., Khalifa N.B., Tekkaya A. (2015). Prediction of roughness after ball burnishing of thermally coated surfaces. J. Mater. Process. Technol..

[6] Amini S., Bagheri A., Teimouri R. (2018). Ultrasonic-assisted ball burnishing of aluminum 6061 and AISI 1045 steel. Mater. Manuf. Process..

[7] Kozlov A., Mordyuk B., Chernyashevsky A. (1995). On the additivity of acoustoplastic and electroplastic effects. Mater. Sci. Eng. A.

[8] Siu K., Ngan A., Jones I. (2011). New insight on acoustoplasticity–ultrasonic irradiation enhances subgrain formation during deformation. Int. J. Plast..

[9] Jerez-Mesa R., Landon Y., Travieso-Rodriguez J.A., Dessein G., Lluma-Fuentes J., Wagner V. (2018). Topological surface integrity modification of AISI 1038 alloy after vibration-assisted ball burnishing. Surf. Coatings Technol..

[10] Huang H., Pequegnat A., Chang B., Mayer M., Du D., Zhou Y. (2009). Influence of superimposed ultrasound on deformability of Cu. J. Appl. Phys..

[11] Liu X., Osawa Y., Takamori S., Mukai T. (2008). Microstructure and mechanical properties of AZ91 alloy produced with ultrasonic vibration. Mater. Sci. Eng. A.

[12] Yao Z., Kim G.Y., Faidley L., Zou Q., Mei D., Chen Z. (2012). Effects of superimposed high-frequency vibration on deformation of aluminum in micro/meso-scale upsetting. J. Mater. Process. Technol..

[13] Ashida Y., Aoyama H. (2007). Press forming using ultrasonic vibration. J. Mater. Process. Technol..

[14] Jin M., Murakawa M. (2001). Development of a practical ultrasonic vibration cutting tool system. J. Mater. Process. Technol..

[15] Moriwaki T., Shamoto E. (1991). Ultraprecision diamond turning of stainless steel by applying ultrasonic vibration. CIRP Ann..

[16] Moriwaki T., Shamoto E. (1995). Ultrasonic elliptical vibration cutting. CIRP Ann..

[17] Brehl D., Dow T. (2008). Review of vibration-assisted machining. Precis. Eng..

[18] Babitsky V., Astashev V., Kalashnikov A. (2004). Autoresonant control of nonlinear mode in ultrasonic transducer for machining applications. Ultrasonics.

[19] Martinez-Gonzalez E., Ramirez G., Romeu J., Casellas D. (2015). Damage induced by a spherical indentation test in tool steels detected by using acoustic emission technique. Exp. Mech..

[20] Jerez-Mesa R., Travieso-Rodriguez J.A., Gomez-Gras G., Lluma-Fuentes J. (2018). Development, characterization and test of an ultrasonic vibration-assisted ball burnishing tool. J. Mater. Process. Technol..

[21] Arnau A. (2004). Piezoelectric Transducers and Applications.

[22] Jerez-Mesa R., Travieso-Rodríguez J.A., Landon Y., Dessein G., Lluma-Fuentes J., Wagner V. (2019). Comprehensive analysis of surface integrity modification of ball-end milled Ti-6Al-4V surfaces through vibration-assisted ball burnishing. J. Mater. Process. Technol..

[23] Ewins D.J. (1984). Modal Testing: Theory and Practice.

[24] Gómez-Gras G., Travieso-Rodríguez J.A., González-Rojas H.A., Nápoles-Alberro A., Carrillo F.J., Dessein G. (2015). Study of a ball-burnishing vibration-assisted process. Proc. Inst. Mech. Eng. Part B J. Eng. Manuf..

